# Undeclared animal species in dry and wet novel and hydrolyzed protein diets for dogs and cats detected by microarray analysis

**DOI:** 10.1186/s12917-018-1528-7

**Published:** 2018-06-27

**Authors:** Rebecca Ricci, Daniele Conficoni, Giada Morelli, Carmen Losasso, Leonardo Alberghini, Valerio Giaccone, Antonia Ricci, Igino Andrighetto

**Affiliations:** 1 Department of Animal Medicine, Production and Health, Viale Dell’Università 16, 35020 Legnaro, Italy; 2 Department of Food Safety, Istituto Zooprofilattico Sperimentale delle Venezie, Viale Dell’Università 10, 35020 Legnaro, Italy

**Keywords:** Pet food, Meat species identification, Novel protein diet, Hydrolyzed protein diet, Mislabeling, Species substitution, Adverse food reaction

## Abstract

**Background:**

Although the European Pet Food Industry Federation (FEDIAF) stated that labels must be accurate and provide detailed information on the ingredients, mislabeling of pet food has been documented by several authors. This phenomenon is of particular concern when related to products used as elimination diets for the diagnosis of adverse food reaction (AFR) in dogs and cats because the presence of undeclared ingredients may negatively interfere with the trial and prevent the veterinarian from making an appropriate diagnosis. The aim of this study was to shed light upon the problem of contamination and mislabeling in both dry and wet novel protein diets (NPDs) and hydrolyzed protein diets (HPDs) using a microarray-based commercial kit which tests for the presence of 19 animal species.

**Results:**

Of the 40 analyzed products (9 dry NPDs, 22 wet NPDs, 6 dry HPDs and 3 wet HPDs), ten presented a content that correctly matched the label, while five did not contain the declared animal species, twenty-three revealed the presence of undeclared animal species, and two had a vague label that did not allow the evaluation of its accuracy. The most frequently contaminants identified in both dry and wet pet foods were pork, chicken and turkey. The presence of undeclared animal species was higher in dry than wet pet foods; furthermore, a lower number of contaminating animal species was identified in HPDs than NPDs (4 vs 10), and a lower number of contaminated HPDs (6 out of 9, 67%) than contaminated NPDs was detected (24 out of 31, 77%). Thirteen out of 14 brands tested presented at least one mislabeled product.

**Conclusions:**

Mislabeling seems to be a widespread issue in pet foods used as elimination diets. Contamination can occur in all types of products used for the purpose, although dry NPDs are the main issue. Due to the high risk of contamination, particular attention should be given to both the selection of raw material suppliers and the production process.

## Background

Mislabeling is an important concern for people who require a controlled diet, and the issue has recently arisen in regard to dog and cat food as well. In a study conducted in the United Kingdom, when testing for the presence of bovine, chicken, porcine and horse DNA in 17 popular wet pet foods obtained from supermarkets, Maine et al. [1] observed that cow, pig and chicken were included in 15 products even when not explicitly stated on their labels. Similarly, when testing a wide range of dog and cat foods (*n* = 52) available in the US market (from both retail and online stores) for the presence of mitochondrial DNA of eight different animal species (i.e. bovine, caprine, ovine, chicken, goose, turkey, porcine, and equine), Okuma and Hellberg [2] found that 38.5% of pet foods were potentially mislabeled because they either contained meat species not declared in the label or did not contain meat species that were. The pet food samples included in the studies by Maine et al. [1] and by Okuma & Hellberg [2] were intended for the maintenance of dogs and cats. A more particular concern has been raised recently regarding the mislabeling of pet food products specifically formulated to contain a single source of protein (novel protein diets, NPDs) [3–5], which along with hydrolyzed protein diets (HPDs) are used as elimination diets for the diagnosis of adverse food reaction (AFR) in dogs and cats. The rationale behind these two dietary approaches is clearly explained in the review by Verlinden et al. [6].

Since dietary trial is the only reliable method to diagnose AFR [7], the presence of undeclared animal species in NPDs and HPDs is a major concern when feeding a food-hypersensitive dog or cat an unpredictably contaminated product because any potentially allergenic protein may preclude significant remission of symptoms and mislead the clinician in diagnosing AFR [4]. Elimination diets, both NPDs and HPDs, are also used in dogs with chronic enteropathies to diagnose food-responsive diarrhea (FRD), antibiotic-responsive diarrhea (ARD) and steroid-responsive disease (SRD) on the basis of the dog’s response to treatment [8, 9].

When considering studies focused on the presence of contaminants in commercial elimination diets, Raditic et al. [3] used an ELISA method to test four over-the-counter venison canine dry foods for the presence of soy, poultry and beef; Ricci et al. [4] analyzed twelve canine dry pet foods used as dietary elimination trials with three zoological classes in mind (i.e. mammal, poultry and fish) via both PCR and a microscopy protocol, whereas in the study by Horvath-Ungerboeck et al. [5], a real time PCR test was used on seven dry and five wet pet foods to identify five animal species (i.e. chicken, turkey, beef, mutton and pork).

Due to the higher stability of DNA molecules compared to proteins when exposed to high temperature, DNA-based protocols seem to be the most reliable methods for the identification of animal species in highly processed foods such as pet food [10].

In the three studies mentioned above, the protocols used for animal species identification were time-consuming and focused on detecting a limited number of animal species in a small number of canine pet food samples, most of which (23 out of 28) were dry [1–5].

The aim of this study was to shed light upon the problem of contamination and mislabeling in dry and wet pet foods used as elimination diets for the diagnosis of canine and feline AFR. Unlike the aforementioned studies, the current investigation adopted a rapid DNA-based microarray for the detection of 19 animal species in a single run. The presence of a contaminant was assumed whenever an animal species not declared in the ingredient list was detected by microarray analysis.

## Methods

### Sample recruitment

A total of 40 pet foods (15 dry and 25 wet) for dogs (*n* = 36) and cats (*n* = 4) produced by 14 different producers were collected from the market. The samples included single batches of 31 NPDs (9 dry and 22 wet) and 9 HPDs (6 dry and 3 wet). Pet foods labels were carefully read to identify every source of protein and fat in the ingredients list. From each pet food, an aliquot of 50 g was sampled and sent to the laboratory for animal species identification. All the analyses were performed in the Food Microbiology laboratory of the Istituto Zooprofilattico Sperimentale delle Venezie (Legnaro, Italy).

### Animal species identification

All samples were treated according to the GeneTop Meat-V kit protocol (GeneTop, Taiwan -R.O.C.). The procedure is an accredited method of the *Istituto Zooprofilattico Sperimentale delle regioni Lazio e Toscana M. Aleandri* [11]. The protocol is based on the extraction of the total DNA fraction, the amplification of specific DNA targets via polymerase chain reaction (PCR), and subsequent DNA microarray assay on solid matrix.

### DNA extraction

An aliquot of 200 mg of either homogenized wet pet food or ground dry pet food was sampled under sterile conditions in duplicate for each product. The samples were processed for DNA extraction using the DNeasy *mericon* food kit (QIAGEN, Hilden, Germany) according to manufacturer instructions. DNA quality and concentration were assessed after extraction by spectrophotometer (SmartSpec™ Plus spectrophotometer, Bio-Rad, USA).

### DNA amplification and microarray assay

The reagents used for amplification and hybridization were all supplied with the GeneTop Meat-V kit (GeneTop, Taiwan - R.O.C.). Briefly, amounts of 22.25 μl of Meat-IV Mix and 0.25 μl of Taq were transferred in sterile PCR tubes kept at low temperature. PCR samples were prepared by adding 2.5 μl of the previously extracted DNA to the reaction mix. PCRs were carried out in a GeneAmp PCR System 9700 (Applied Biosystem, USA) using the following PCR protocol: 2 min at 94 °C followed by 30 cycles of 94 °C for 30s, 60 °C for 30s, and 72 °C for 30s. A final elongation step was performed at 72 °C for 10 min. The amplified samples were then stored at − 20 °C until use.

### Microarray analysis

The microarray plate was designed to detect the presence of 19 animal species: bovine, buffalo, cat, chicken, dog, donkey, duck, hare, fish, goat, goose, horse, mice, porcine, poultry, rabbit, rat, sheep, turkey.

The protocol used for microarray identification was supplied by the manufacturer (GeneTop, Taiwan - R.O.C.). Briefly, the amplicons obtained by PCR were denatured by heating to 95 °C for 5 min and 30 s, then placed on ice. The pre-warmed (to room temperature) HA Buffer was pipetted in advance in each Meat-V Chip well and the liquid was spread over the surface of every chip. A 5 μl amount of the denatured amplicons was loaded in each well, and then the biochip was covered with adhesive film. The biochip was then incubated at 50 °C for 40 min in the hybridization shaker (1000 rpm). The hybridization liquid was discarded and the wells were washed three times using 200 μl of Wash buffer. The Blocking reagent was prepared with a StrepAP and B buffer ratio of 1:1000, and a total 100 μl of the mixture was poured into each well and incubated for 20 min at 50 °C. The biochip was washed 3 times with 200 μl of Wash buffer and then 200 μl of C buffer was added and discarded. The detection buffer composed of 2 μl of NBT/BCIP mixed with 98 μl of C buffer (ratio 1:50) was added to each well and the biochip was placed in the dark for 7 min. The detection buffer was discarded and the chip was rinsed with tap water twice. The dried biochip was then read using the GeneTop reader (GeneTop, Taiwan - R.O.C.) and by naked eye. According to the manufacturer, the test sensibility is 0.1%. The results were recorded in a Microsoft Excel spreadsheet (Microsoft Corporation, Redmond, WA).

## Results

The following animal species were detected in at least one sample of the pet foods tested: bovine, chicken, duck, fish, goat, goose, horse, pork, poultry, rabbit, sheep and turkey. The following species instead were not detected in any sample: buffalo, cat, dog, donkey, hare, mice and rat.

The results obtained by DNA-based microarray assay are shown in Table 1.

**Table 1:** ID number, samples identification (brand, type of elimination diet and addressed animal species, declared animal protein source, protein and fat sources of animal origin as stated in the ingredients list) and animal species DNA identified in dry and wet novel protein diets (NPDs) and hydrolyzed protein diets (HPDs) by microarray analysis

Sample ID	Brand ID	Product type	Declared protein sources	Protein sources in ingredients list	Fat sources in ingredients list	Animal species DNA detected in the samples											
						Pork	Bovine	Horse	Fish	Chicken	Turkey	Duck	Goose	Sheep	Goat	Rabbit	Poultry
Dry pet foods																	
1	A	Dog- NPD	Duck	Dehydrated duck meat		+	+	–	+	+	+	+	+	–	–	–	+
2	B	Dog- NPD	Duck	Dehydrated duck meat		+	+	–	–	+	+	–	–	+	–	–	+
3	A	Dog- NPD	Horse	Dehydrated horse meat		+	+	+	–	+	+	–	–	–	–	–	+
4	B	Dog- NPD	Horse	Dehydrated horse meat		+	–	+	+	+	–	–	–	+	–	–	+
5	C	Dog- NPD	Lamb	Lamb meat meal, hydrolyzed lamb meat, hydrolyzed liver	Poultry fat	+	+	+	+	+	+	+	–	+	–	–	+
6	D	Dog- NPD	Salmon	Fresh salmon, hydrolyzed salmon, salmon meal	Animal fat, fish oil	+	+	–	+	+	+	–	–	+	–	–	+
7	A	Dog- NPD	Salmon	Hydrolyzed salmon meal		+	+	+	+	+	+	+	–	–	–	–	+
8	E	Dog- NPD	Swine	Animal proteins, ham	Purified swine fat, fish oil	+	+	–	–	+	+	–	–	+	–	–	+
9	B	Dog- NPD	Venison	Hydrolyzed venison		+	+	+	–	+	+	–	–	+	+	–	+
10	F	Dog- HPD	Hydrolyzed chicken	Hydrolyzed chicken liver		+	–	–	–	+	+	–	–	–	–	–	+
11	G	Dog- HPD	Hydrolyzed chicken	Low molecular weight hydrolyzed feather	Fish oil, animal fats	+	–	–	–	+	+	–	–	–	–	–	+
12	G	Dog- HPD	Soy	Hydrolyzed chicken liver	Animal fats, fish oil	+	–	–	–	+	+	–	–	–	–	–	+
13	G	Cat- HPD	Soy	Hydrolyzed chicken liver	Animal fats, fish oil	+	+	–	+	+	+	–	+	–	–	–	+
14	H	Dog- HPD	Soy		Fish oil	–	–	–	–	–	–	–	–	–	–	–	–
15	H	Cat- HPD	Soy	Hydrolyzed offal	Animal fat, fish oil	+	–	–	–	+	–	–	–	–	–	–	+
Wet pet foods																	
16	G	Cat- NPD	Chicken	Chicken liver and meat	Fish oil	–	–	–	–	+	–	–	–	–	–	–	+
17	A	Dog- NPD	Duck	Duck meat		+	+	–	–	–	+	–	–	–	–	–	+
18	B	Dog- NPD	Duck	Duck		–	–	–	–	–	–	+	–	–	–	–	+
19	I	Dog- NPD	Duck	Fresh duck		+	+	–	–	+	+	–	–	–	–	–	+
20	J	Dog- NPD	Duck	Duck meat		+	–	–	–	+	–	+	–	–	–	–	+
21	K	Dog- NPD	Goat	Goat heart, lung, liver, meat, goat meat broth	Salmon oil	–	–	–	–	–	–	–	–	+	+	–	–
22	A	Dog- NPD	Horse	Horse meat		+	+	+	–	+	–	–	–	–	–	–	–
23	B	Dog- NPD	Horse	Horse		–	–	+	–	–	–	–	–	–	–	–	–
24	L	Dog- NPD	Horse	Horse meat		–	–	+	–	–	–	–	–	–	–	–	–
25	K	Dog- NPD	Horse	Horse heart, lung, liver, stomach, meat, horse meat broth	Salmon oil	–	–	+	–	–	–	–	–	–	–	–	–
26	D	Dog- NPD	Salmon	Salmon		+	–	–	+	–	–	–	–	–	–	–	–
27	A	Dog- NPD	Salmon	Fish		–	–	–	+	–	–	–	–	–	–	–	–
28	F	Dog- NPD	Salmon	Fish and fish by-products	Oils and fats	+	–	–	–	+	+	–	–	–	–	–	+
29	M	Dog- NPD	Salmon	Salmon		+	+	–	+	+	+	–	–	+	–	+	+
30	N	Dog- NPD	Swine	Swine meat		+	–	–	–	+	+	–	–	–	–	–	+
31	B	Dog- NPD	Swine	Pork		+	–	–	–	–	–	–	–	–	–	–	–
32	K	Dog- NPD	Turkey	Turkey heart, liver, meat, throat, stomach, turkey meat broth	Salmon oil	–	–	–	–	+	+	–	–	–	–	–	+
33	B	Dog- NPD	Venison	Venison		+	–	–	–	–	–	–	–	–	–	–	–
34	C	Dog- NPD	Venison	Venison and deer offal, deer broth		+	+	+	–	+	–	–	–	+	–	–	+
35	F	Dog- HPD	Hydrolyzed chicken	Hydrolyzed chicken liver		–	–	–	–	+	–	–	–	–	–	–	–
36	F	Cat- HPD	Hydrolyzed chicken	Hydrolyzed chicken liver		–	–	–	–	+	–	–	–	–	–	–	–
37	G	Dog- HPD	Soy	Hydrolyzed poultry liver	Oils and fats	+	–	–	–	–	–	–	–	–	–	–	–
38	I	Dog- NPD	Lamb	Lamb meat		+	–	–	–	+	+	–	–	+	–	–	+
39	I	Dog- NPD	Turkey	Turkey meat		+	–	–	–	+	+	–	–	+	–	–	+
40	I	Dog- NPD	Chicken	Chicken meat		+	+	–	–	+	+	–	–	–	–	–	+

**Fig. 1:**
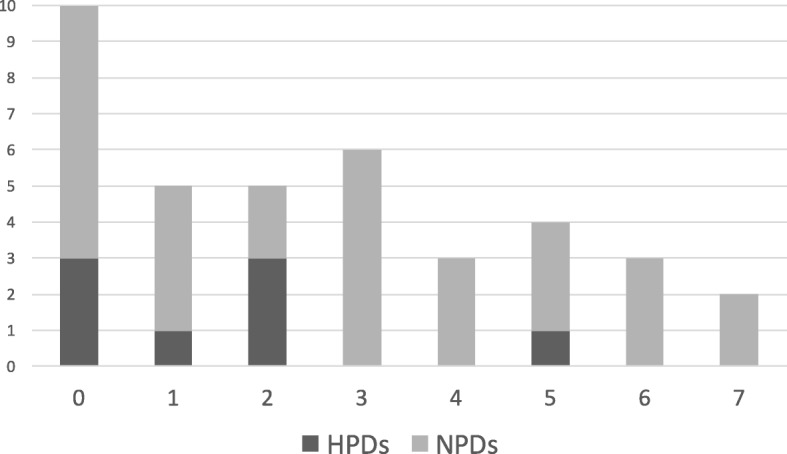
Number of hydrolyzed protein diets (HPDs) and novel protein diets (NPDs) presenting 0 to 7 undeclared animal species detected by microarray analysis

The composition of 38 out of 40 samples was indicated on the label by a list of ingredients that clearly allowed the identification of the animal species included, whereas the remaining two product labels used terms like ‘animal proteins’ in sample ID 8 and ‘hydrolyzed offal’ in sample ID 15 that did not permit such identification. Despite their positivity for two and five animal species respectively, it was therefore impossible to state the inadequacy of these two product labels. For all the other 38 pet foods, label adequacy was evaluated as follows. Ten samples (one dry and nine wet; seven NPDs and three HPDs) were labeled correctly because only the DNA of the protein source stated in the ingredient list was detected (sample IDs: 14, 16, 18, 23–25, 27, 31, 35, 36). Five samples (one dry and four wet; four NPDs and one HPDs) were mislabeled because the DNA of the protein source stated in the ingredient list was not detected (sample IDs: 2, 17, 19, 28, 37); in particular, in three samples (ID 2,17,19) duck was not detected, in sample ID 28 the salmon declared on the label did not trigger positivity for fish, and in sample ID 37 the hydrolyzed poultry liver specified on the label did not trigger the positivity expected either for chicken, turkey, or even poultry. In the remaining 23 samples, the DNA of the protein source stated in the ingredient list was detected, but the DNA of one to seven other protein sources not listed on the label was detected as well (Fig. 1).

Table 2 shows the number of dry and wet pet foods positive to an animal species not declared on the label.

**Table 2:** Number and percentage (%) of dry and wet pet foods in which animal species not listed on the label were detected

	DRY pet foods		WET pet foods	
Animal species detected by the microarray	Number of pet foods in which the animal species was not listed in the label	Number and % of pet foods in which such unlisted species was detected	Number of pet foods in which the animal species was not listed in the label	Number and % of pet foods in which such unlisted species was detected
Sheep	12	4 (33.3%)	24	4 (16.7%)
Duck	11	2 (18.2%)	21	0 (0%)
Bovine	13	8 (61.5%)	25	6 (24%)
Goat	13	1 (7.7%)	24	0 (0%)
Goose	15	1 (6.7%)	24	0 (0%)
Horse	11	3 (27.3%)	21	1 (4.8%)
Pork	13	12 (92.3%)	23	13 (56.5%)
Chicken^a^	9	8 (88.9%)	20	10 (50%)
Poultry^b^	7	6 (85.7%)	14	5 (35.7%)
Fish	11	4 (36.4%)	21	0 (0%)
Turkey	13	11 (84.6%)	23	7 (30.4%)

^a^chicken or hydrolyzed chicken was declared on the label

^b^chicken, turkey, hydrolyzed chicken or duck or goose was declared on the label

The contaminants most frequently identified by the microarray kit in dry pet foods were pork, chicken, poultry and turkey, followed by bovine, fish and ovine, then horse; on the other hand, the least common contaminants were duck and goat. Regarding wet pet foods, pork was the most common contaminant, followed by chicken, poultry, turkey, bovine and sheep; the least common contaminant was horse, while fish, duck and goat were not detected in these samples.

Since the microarray used in this study lacked a deer DNA detection well, it was impossible to both confirm the presence of venison in the three samples that listed such species on the label and identify venison contamination in the others.

In general, one dry sample out of 13 and nine wet samples out of 25 did not show any DNA contamination from animal species not listed on the label.

Fewer animal species not listed on the label were identified in HPDs than in NPDs (4 vs 10, Fig. 1); moreover, a higher number of contaminated HPDs (6 out of 9, 67%) than contaminated NPDs was detected (24 out of 31, 77%). More details are reported in Table 3.

**Table 3:** Number and percentage (%) of hydrolyzed protein diets (HPDs) and novel protein diets (NPDs) in which animal species not listed on the label were detected

	HPDs		NPDs	
Animal species detected by the microarray	Number of pet foods in which the animal species was not listed in the label	Number and % of pet foods in which such unlisted species was detected	Number of pet foods in which the animal species was not listed in the label	Number and % of pet foods in which such unlisted species was detected
Sheep	8	0 (0%)	28	8 (28.6%)
Duck	8	0 (0%)	24	2 (8.3%)
Bovine	8	1 (12.5%)	30	13 (43.3%)
Goat	8	0 (0%)	29	1 (3.4%)
Goose	8	0 (0%)	29	1 (3.4%)
Horse	8	0 (0%)	24	4 (16.7%)
Pork	8	5 (62.5%)	28	20 (71.4%)
Chicken^a^	1	0 (0%)	28	18 (64.3%)
Poultry^b^	1	0 (0%)	20	11 (55%)
Fish	8	1 (12.5%)	24	3 (12.5%)
Turkey	8	4 (50%)	28	14 (50%)

^a^chicken or hydrolyzed chicken was declared on the label

^b^chicken, turkey, hydrolyzed chicken or duck or goose was declared on the label

In HPDs, the most common contaminants detected by microarray analysis were pork, turkey, bovine and fish. Pork, chicken, poultry, turkey, bovine and sheep were the most common contaminants in NPDs.

With the exception of brand L, at least one mislabeled product was identified in all the brands considered (Table 4).

**Table 4:** Number of mislabeled and correctly labeled dry and wet pet foods divided by brand

	DRY pet foods		WET pet foods	
Brand ID	Mislabeled	Correctly labeled	Mislabeled	Correctly labeled
A	3	0	2	1
B	3	0	1	3
C	1	0	1	0
D	1	0	1	0
E	1	0	0	0
F	1	0	1	2
G	3	0	1	1
H	1	1	0	0
I	0	0	4	0
J	0	0	1	0
K	0	0	2	1
L	0	0	0	1
M	0	0	1	0
N	0	0	1	0

## Discussion

As stated by the European Pet Food Industry Federation (FEDIAF [12]), pet food labels must be accurate and all ingredients unequivocally identified. This is particularly important for pet foods formulated as elimination diets for the purpose of diagnosing an adverse food reaction in dogs or cats because the accuracy of the label and correspondence with the ingredients actually included in the formula assume paramount importance in the success of the trial.

In this study, we performed microarray analysis on a total of 40 products formulated for dietary elimination trials in order to investigate possible contaminants and mislabeling. The ingredient list of samples ID 8 and ID 15 provided vague indications of meat content (i.e. ‘animal proteins’ and ‘hydrolyzed offals’, respectively), thereby precluding the identification of the animal species listed on label by reading alone. Therefore, any animal species may have been included, and although microarray analysis of these two products detected more than one species, the non-adequacy of the products cannot be stated with more accuracy. European Regulation (EC) No 767/2009 [13] allows categories of ingredients to be listed on pet food labels, and many pet food products take advantage of this option. However, vague ingredient lists should not be acceptable in pet foods intended for the diagnosis of adverse food reactions. All the remaining 38 products had detailed ingredient lists, and meat species were clearly reported in their labels.

According to microarray results, only 25% of the products analyzed were suitable for effective AFR diagnosis. Although these findings demonstrate that producing an uncontaminated pet food is possible, three out of four commercial elimination diets would not be useful in allowing the clinician to obtain precise AFR diagnosis.

The five mislabeled samples whose declared animal species were not detected by the micro-array analysis can be construed as a commercial fraud punishable by law with additional complications for producers other than leading to mistaken diagnosis for food allergic animals. Samples listed as containing duck (ID 2,17,19) were instead positive to both poultry and chicken and/or turkey, and this may be considered intentional substitution with cheaper ingredients of avian origin. Commercial frauds in pet foods were reported in a previous study by Okuma and Hellberg [2] where six products were not found to contain the meat species declared on the front of the package.

On the other hand, the consequences of including undeclared animal species in elimination diets have already been presented elsewhere [4]. The presence of one to multiple animal species not declared on the label of elimination diets was demonstrated by a PCR-based approach in previous studies [4, 5], whereas the microarray analysis performed here was able to identify up to 19 animal species in a single run.

The most common animal species not declared on the labels of both dry and wet pet foods were pork, chicken and turkey. In the study by Horvath-Ungerboeck et al. [5], the most common contaminants were beef (positive in 8/12 samples) and pork (positive in 6/12 samples). Since pork, poultry and beef are the animal species most commonly slaughtered in Europe for human consumption [14], they are the ones that provide the largest amount of animal by-products to pet food manufacturers. Given that their meat is cheaper than that of other species like duck, venison, and goat, economic reasons may underline the frequent detection of these animal species as contaminants, as suggested by Okuma and Hellberg [2]. This may also be the reason why goat and duck were the contaminating animal species least frequently identified in this study. These species were not investigated in the recent study by Horvath-Ungerboeck et al. [5], which considered only five animal species (chicken, turkey, beef, mutton and pork).

Our results showed that undeclared animal species were more commonly found in dry than wet pet foods. This is in contrast with the results by Okuma and Hellberg [2] in which the rate of mislabeled wet foods (*n* = 12/16) was higher than that of mislabeled dry pet foods (2/17). In a study by Ricci et al. [4] in which only dry pet foods were included, no comparison between dry and wet diets was possible, whereas Horvath-Ungerboeck et al. [5] analyzed 8 dry complete diets and 4 canned (2 complete and 2 complementary) products, in which 6 dry (75%) and 3 canned (75%) resulted mislabeled. One possible reason for the more frequent contamination observed in dry products could be that the technological process does not allow thorough cleaning between the production of consecutive batches. Another reason might regard the quality of the incoming raw materials: the dehydrated meat meals used in dry pet food production may be contaminated with other different meat meals during production, transport or storage. Whether this is a more significant possibility for meat meals than the meat-derivatives used for wet pet food production remains conjecture.

HPDs have been investigated less in previous studies on contamination in elimination diets: there was only 1 HPD in the 12 elimination examined in the study by Ricci et al. [4] and only 2 HPDs in the 12 pet foods studied by Horvath-Ungerboeck et al. [5]. In the present study that includes 9 HPDs, stronger conclusions can be drawn. It may therefore be stated that fewer contaminating animal species were identified in HPDs than in NPDs and a lower number of contaminated HPDs was detected than NPDs. Even hypothesizing that producers take extra care when producing HPDs, contamination of hydrolysed raw material at their production site (and therefore prior to delivery to the pet food plant) or during transportation cannot be excluded. The present results demonstrated that even if pork was not mentioned in their ingredient list, five HPDs were positive to swine DNA. One of these five products labels stated “hydrolyzed animal offals” in the ingredient list, which does not exclude the presence of pork; however, the remaining four were chicken- and/or soy-based products. There is no way of knowing whether the swine DNA detected was derived from hydrolysed or non-hydrolysed swine protein or even swine fat; this notwithstanding, these samples were not listed as containing swine DNA and this can be construed as contamination. Since HPDs are used as elimination diets for the ARF diagnosis, contamination implies that the same consequences hypothesized for contaminated NPDs be taken into consideration. This means that both HPDs and NPDs may pose problems in the diagnosis of AFR.

Although only few products were collected for some brands (only one product for four brands and only two products for three brands), it may be stated that as observed in 13 of the 14 brands tested, mislabeling is a widespread phenomenon.

## Conclusions

In conclusion, microarray analysis suggested that in a survey of 38 pet food products formulated for the diagnosis of AFR in dogs and cats, 3 out of 4 were contaminated with one to seven animal species not listed on the label. This appears to be an issue that regards both novel and hydrolyzed protein diets, with a higher number of contaminating animal species being detected in the former. Contamination also seems to occur more predominantly in dry than wet products, regardless of pet food producer. Particular attention should be given to both the production process due to the high risk of cross-contamination and the selection of raw material suppliers. Bearing this in mind, these results reflect the contamination of only the batches collected here, and therefore the mislabeling of a specific product cannot be generalized either to the respective producer’s previous or future productive lots. Importantly, this study demonstrates that the production of uncontaminated products is possible, as confirmed by the ten pet foods (26%) that analysis found to be correctly labeled. When an adverse food reaction is suspected, a home-made elimination diet may be a reasonable alternative to commercial products because it allows a stricter control of the ingested ingredients and highly reduces the risk of contaminants that pose risks to the correct diagnosis of AFR.

## Abbreviations

AFR: Adverse food reaction
HPDs: Hydrolyzed protein diets
NPDs: Novel protein diets
