# Correction to: Undeclared animal species in dry and wet novel and hydrolyzed protein diets for dogs and cats detected by microarray analysis

**DOI:** 10.1186/s12917-021-02779-z

**Published:** 2021-02-15

**Authors:** Rebecca Ricci, Daniele Conficoni, Giada Morelli, Carmen Losasso, Leonardo Alberghini, Valerio Giaccone, Antonia Ricci, Igino Andrighetto

**Affiliations:** 1 Department of Animal Medicine, Production and Health, Viale Dell’Università 16, 35020 Legnaro, Italy; 2 Vetekipp S.r.l., via della Croce Rossa 112, 35129 Padova, Italy; 3 Department of Food Safety, Istituto Zooprofilattico Sperimentale delle Venezie, Viale Dell’Università 10, 35020 Legnaro, Italy

**Correction to: BMC Vet Res 14, 209 (2018)**

**https://doi.org/10.1186/s12917-018-1528-7**

The original article [1] published does not declare the full affiliation and competing interests statement for author Dr. Rebecca Ricci.

**Affiliations**

The full affiliation for Rebecca Ricci should be listed as:

Department of Animal Medicine, Production and Health, Viale Dell’Università 16, 35020 Legnaro, Italy; Vetekipp S.r.l., via della Croce Rossa 112, 35129 Padova, Italy

**Competing interests**

At the time the paper was published, Dr. Rebecca Ricci was affiliated to Vetekipp S.r.l., an Italian company that commercialize a software to formulate balanced home-made diets for dogs and cats, and vitamin and mineral supplements to balance home-prepared diets for dogs and cats. Dr. Ricci desires to declare her affiliation to Vetekipp S.r.l. for transparency, however it must be stated that being part of Vetekipp did not influence Dr. Ricci’s interpretation of the present study’s data, neither did it bias the presentation of information in the manuscript. Moreover, at the time the study was planned and laboratory analyses were run Vetekipp S.r.l. was not established yet; Vetekipp S.r.l. did not sponsored or funded this research study. All other authors declare no competing interests.
